# Experimental and Statistical Study of the Fracture Mechanism of Sn96.5Ag3Cu0.5 Solder Joints via Ball Shear Test

**DOI:** 10.3390/ma15072455

**Published:** 2022-03-26

**Authors:** Kun Liang, Yuexing Wang, Zhigang He

**Affiliations:** 1 Institute of Electronic Engineering, China Academy of Engineering Physics, Mianyang 621999, China; soceity@mail.nwpu.edu.cn; 2 Microsystem and Terahertz Research Center, China Academy of Engineering Physics, Chengdu 610200, China; 3 The Center of Metrology and Testing, China Academy of Engineering Physics, Mianyang 621999, China; phyhezhigang@163.com

**Keywords:** Sn96.5Ag3Cu0.5, ball shear test, statistical analysis, SEM image, fracture mechanism, reliability

## Abstract

Ball shear testing is an efficient approach to investigate the mechanical reliability of solder joints at the structural level. In the present study, a series of low-speed ball shear tests were conducted to study the deformation and fracture characteristics of Sn96.5Ag3Cu0.5 solder joints at continuous speeds from 10 μm/s to 200 μm/s. In order to account for randomness, the quantity of tests was repeated for each shear rate. The relationship between mechanical properties and shear speeds was calculated in detail via effective statistical analysis. In addition, by utilizing SEM imaging and ingredient analysis the interfacial effect and fracture mechanism of solder balls were obtained and their fracture mode classified into two types, viz., bulk fracture and interface fracture. Furthermore, by means of statistical analysis and approximate calculation it was proven that bulk fracture balls have greater adhesive powers and reliability compared with interface fracture balls.

## 1. Introduction

Due to human health and environmental concerns the use of lead-free solder materials, especially Sn-based solder alloys, have been widely used in interconnecting structures in the microelectronic packaging industry [1,2,3,4,5]. However, Sn-based materials exhibit obvious viscoplasticity at room temperature due to their low melting point, such that room temperature can be considered a ‘high’ temperature [6]. In addition, when compared with traditional lead-containing solders the reliability and stability features of these lead-free solder alloys under complex loadings, such as thermal cycling and mechanical stresses, are not quite clear. Furthermore, given the trend toward the miniaturization of electronic devices, the typical dimensions of solder joints has been decreased to several microlengths [7], at which solder joints show obvious size effects and anisotropy [8,9]. Taking all these factors into consideration, it is recognized that how to intuitively and rapidly estimate the strain strength and reliability levels of these high density packaged lead-free solders represents an important research direction.

In the context of in situ mechanical test approaches for solder joints, the most universal experimental method is ball shear testing. Its popularity is derived from its simple testing procedure and explicit experimental mechanism [10,11]. Although a great deal of research based on this experimental method has been conducted in the field of BGA solders, these research works mainly focus on solder ball materials and manufacturing processes. Their major task has been to distinguish the shear stress characteristics of tested solder balls under various different conditions, including alloy element proportions [2,12,13,14,15], manufacturing processes [16,17], solder ball dimensions [18,19], aging time [18,20], surface finish materials [1,21,22], and more. These research efforts have provided effective support in the procedures of material proportioning and manufacturing process design for highly reliable and stable lead-free solders.

However, particular studies concentrating on ball shear test speeds are relatively lacking. In addition, there are numerous problems in prior works concerning test speeds. First, exorbitant speeds and overlarge speed intervals are usually applied in ball shear experiments. Second, insufficient experimental data analysis has led to a great deal of randomness in the results. Finally, the weak relationship between test speeds and ball fracture characteristics brings about difficulties in utilization in actual scene analysis and finite element simulations. All these problems tend to make the prior works invalid in describing the authentic fracture and degeneration appearance of products under complex thermal and mechanical loadings.

In the current study, large numbers of low-speed ball shear tests based on a specific Sn96.5Ag3Cu0.5 solder structure were conducted at continuous speeds from 10 μm/s to 200 μm/s. Each test was repeated fifteen times in order to investigate the randomness of the mechanical properties of the real solder structure. Contrary to traditional testing procedures, a full elasticity–plasticity deformation process was obtained during our experiments. By utilizing statistical analysis methods, the relationship between mean peak force and shear speed was calculated in detail. In addition, interfacial effects and fracture mechanisms were obtained for the fracture cross sections of all tested samples and classified into types via SEM imaging and ingredient analysis. We believe that a clear bridge has been constructed here between the viscoplastic properties of the solder and the in situ ball shear test experiment.

## 2. Materials and Methods

### 2.1. Ball Shear Specimens and Test Setup

**Figure 1:**
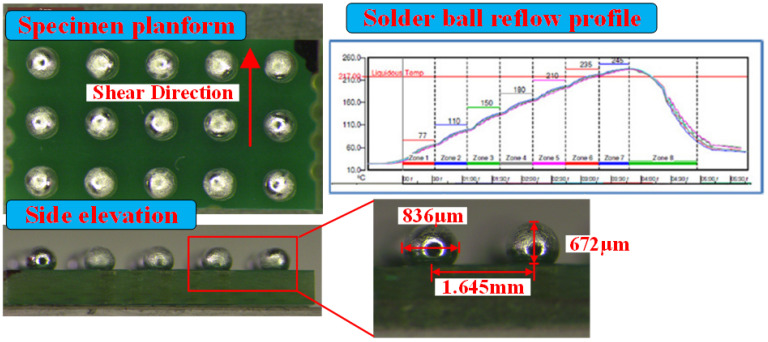
Views of solder ball specimen and the reflow profile.

A category of package level-mounted specimens with lead-free Sn96.5Ag3Cu0.5 ball grid array (BGA) solders bonded on printed circuit boards (PCB) was prepared for ball shear testing. The PCBs were fabricated with FR-4 glass epoxy substrate and a layer of Solder Mask Defined (SMD) Cu padding. A thin film of Ni was plated on the Cu pad to act as a barrier layer in the reflow process for the purpose of preventing the formation of intermetallic compounds between Cu and Sn and improving solder reliability. After solder ball implanting, the thermal reflowing process was conducted for the specimens; the reflow profile utilized here is shown in Figure 1. In this profile, the peak temperature was about 235 °C and the maintaining time above 217 °C was almost 60 s. For each specimen, fifteen solder balls with equivalent dimensions were surface-mounted onto the Cu pads. The average diameter and height of the reflowed solder balls were 836 μm and 672 μm, respectively. In addition, the mean distance between two adjacent balls was 1.645 mm. A specimen plan-form view and a side elevation view along with their respective dimensions are shown in Figure 1.

**Figure 2:**
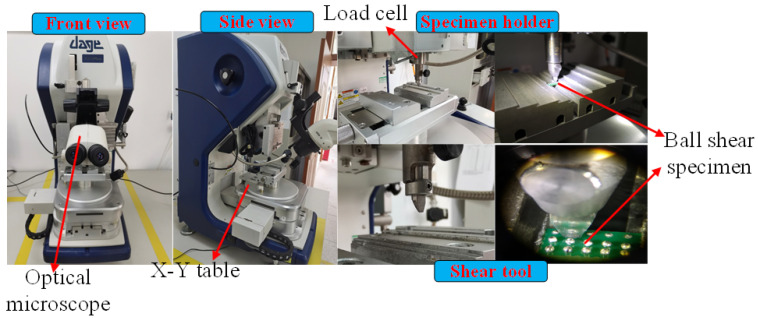
Ball shear test equipment, testing fixture, and shear tool.

The solder ball shearing test was conducted using a Model 4000 Plus Micromechanical Shear Stress Tester manufactured by Dage from the UK. Figure 2 shows the appearance and detailed views of the experimental setup as well as the testing fixture and the ball shear specimens in the actual test procedures. In this equipment, the shear tool is an instrument that directly touches the solder balls and load stresses on the target position. A micrometer actuated X-Y table is used to control the sample holder planar position and the alignment state of the specimen with the shear tool. The relative position and distance between the specimen and the shear tool are commonly checked before testing using the optical microscope. A shear tool with the parameter of 5 kg was employed in this experiment according to the characteristics of the solder samples. The shear tool clearance was set at ~50 μm. The shear load, displacement, and trend of the solders were measured by the load cell attached to the shear tool. Through the ball shear speed setting in the digital control software precision of up to 1 μm/s speed can be achieved in the shearing test. Shear forces and displacements are recorded simultaneously by the software.

### 2.2. Test Procedure

In order to study the effect of shear rates on the Sn96.5Ag3Cu0.5 solder ball deformation features and the relationship between shear load and displacement, twelve continuous low shear speeds ranging from 10 μm/s to 200 μm/s were chosen for this experiment. In addition, fifteen ball samples with almost the same position and dimensions were selected for testing at each shear rate. In every specific experiment, the shear force was loaded by the shear tool from one side of the solder ball to the opposite side with a clearance of 50 μm to the substrate. The shear forces and displacements along with their corresponding time variable data were measured and collected during the shear test, allowing that the force–displacement curves to be determined. Following the experiments, optical and scanning electron microscopy (SEM) examination was conducted on the sheared specimens in order to identify and observe their failure and deformation features as well as the distribution of chemical elements on the fracture surfaces.

## 3. Results and Discussion

### 3.1. Ball Shear Tests at Different Speeds

**Figure 3:**
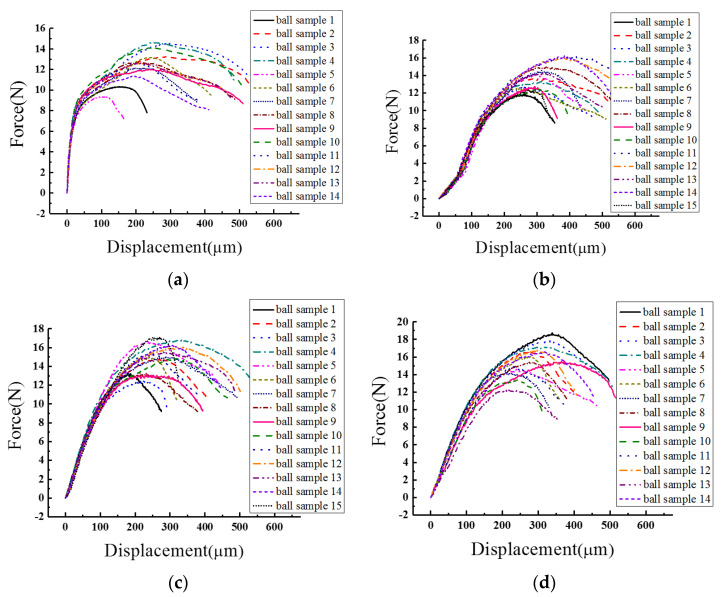
Force−displacement result curves at four representative shearing speeds: (**a**) 20 μm/s; (**b**) 80 μm/s; (**c**) 150 μm/s; and (**d**) 200 μm/s.

Yield strength data on similar solder balls under different shearing load conditions were obtained by setting diverse shear test rates. In order to achieve more precise and abundant force changing rules data, twelve relatively low shear test speeds (10 μm/s to 100 μm/s, with a per 10 μm/s interval increase, along with 150 μm/s and 200 μm/s) were chosen in this experiment. Fifteen repeated tests were then conducted for each shear test rate. Figure 3 shows the force–displacement result curves at four representative shearing speeds, viz., 20 μm/s, 80 μm/s, 150 μm/s, and 200 μm/s.

As shown in Figure 3, the entire set of solder ball samples experienced a transformation process from elastic deformation to plastic deformation. The plastic deformation force of the samples all suffered a decline to different extents after reaching the peak value, namely, the yield strength limitation of the solder material. Different ball samples at each shear test speed show great consistency in the elastic deformation stage, with consistent rates over 90%. However, in the plastic deformation stage there are huge yield strength differences among solder ball samples. The average discrepancy of peak force values among samples in one test rate is approximately 4 N to 6 N. In addition, it is observed that material extendibility varies greatly among ball samples, which induces a difference in the descending delay time after arriving at the peak force.

**Figure 4:**
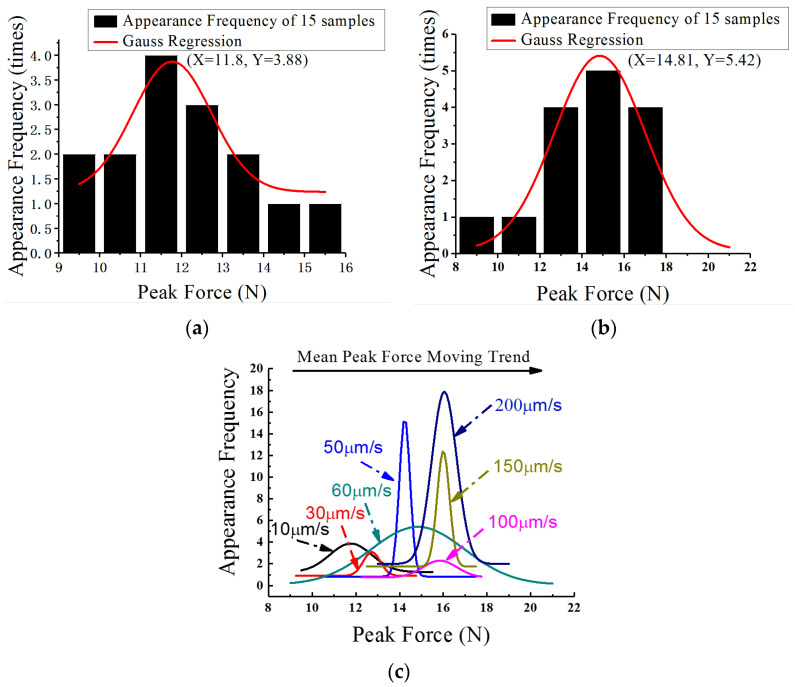
Peak force distributions of typical speed tests and Gaussian regression curves: (**a**) 10 μm/s; (**b**) 60 μm/s; (**c**) seven different speeds.

It can be seen via the statistical analysis of the peak force data in each shear test speed that all the peak force values of the fifteen samples at each rate approximately conform to a Gaussian distribution. The peak force distributions of several typical speed tests and their corresponding Gaussian regression curves are shown in Figure 4.

**Figure 5:**
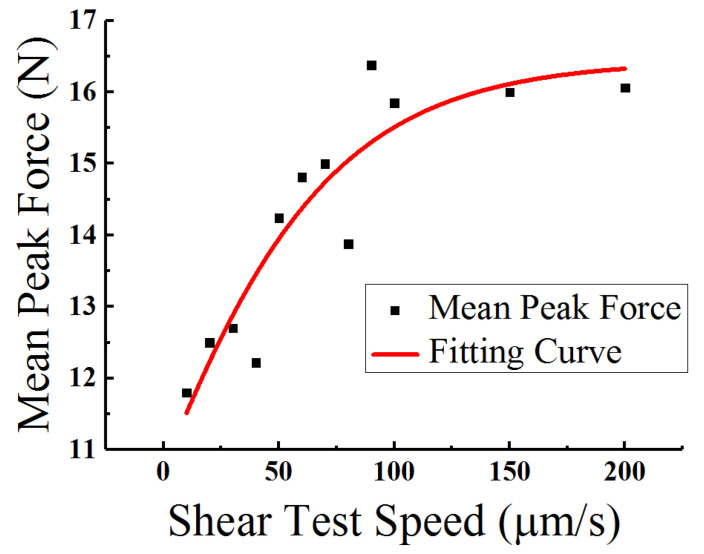
The mean peak force values for all twelve speeds, with fitting curve.

Figure 4a,b shows the apparent frequency distributions of peak force values in the shearing test speeds at 10 μm/s and 60 μm/s, respectively. It is obvious that they all satisfy the characteristics of Gaussian distribution. As shown in the figures, the peak force abscissa of the Gaussian regression statistical analysis curve at 10 μm/s is 11.8 N and 14.81 N at 60 μm/s. According to Figure 4c, at rates of less than 100 μm/s, the peak value abscissa of the Gaussian regression curves (which are the average maximum forces in specific shearing tests as well) gradually increase along with the increment of shear speed. Then, they rapidly become almost steady after the shear rate exceeds 100 μm/s. The mean peak force values of different shear speeds along with the fitting curve are shown in Figure 5. The approximation equation is

(1) $$F_{p}=\frac{a}{1+b\cdot e^{-kv}}$$

where $F_{p}$ represents the mean peak force, v is the shear test speed, and a, b, and k are the fitting factors, with their typical values being 16.44, 0.53, and 0.02, respectively.

As shown in Figure 5, the relationship between mean peak forces and shear speeds can be approximately described by Equation (1). The mean peak forces increase sharply from 12 N to 16 N along with the increment of shear speeds in the range of 10 μm/s to 100 μm/s. After exceeding 100 μm/s, the corresponding peak force values become more steady at about 16 N. This relationship between shear force and speed coincides with previous research on Sn-rich materials [23]. It has been previously reported that the Young’s Modulus and tensile strength of solders increases with the increment of strain rate. Theoretically, the plastic deformation ability of Sn-rich materials is determined by the generating and moving capabilities of dislocations in the strain loading process. The quantity of dislocations that need to migrate simultaneously would then increase sharply with the increment of the strain loading velocity, which could cause the moving capability of dislocations and the plastic deformation ability to decrease. This could lead to an enlargement in the plastic deformation force and yield strength required to generate an equivalent deformation as compared with low speeds. Thus, this represent a good explanation for the developing trend between shearing forces and testing speeds.

### 3.2. Ball Fracture Mechanism Analysis

**Figure 6:**
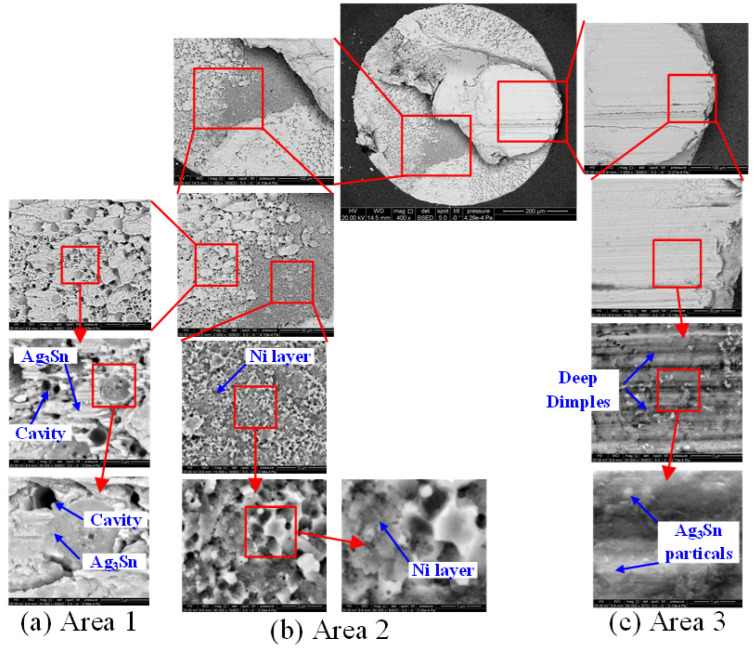
Typical ball SEM image and its partial areas.

Following the above experiments, Scanning Electron Microscopy (SEM) detection was conducted for each solder ball fracture plane. A typical ball image and its partial area figures are exhibited in Figure 6.

According to Figure 6, there are three main different areas on a typical ball fracture surface. Among these, area 1 consists of Ag_3_Sn and numerous cavities, area 2 is the Ni contact layer, and area 3 is a compact Ag_3_Sn material layer. In the ball shear experiment, these three areas represent three different fracture status. Commonly, area 1 represents the largest portion of the fracture plane. In this area, the cracking position is extremely close to the contact layer. In prior research, the cavity growth phenomenon has been attributed to the decohesion of Sn-rich dendrites and the morphology of finer Ag_3_Sn particles [24]. Area 2 indicates complete solder ball stripping, and Ag_3_Sn rarely exists here. In area 3 the tearing location is in the body of the solder ball, and a mass of Ag_3_Sn material is left on the surface.

**Figure 7:**
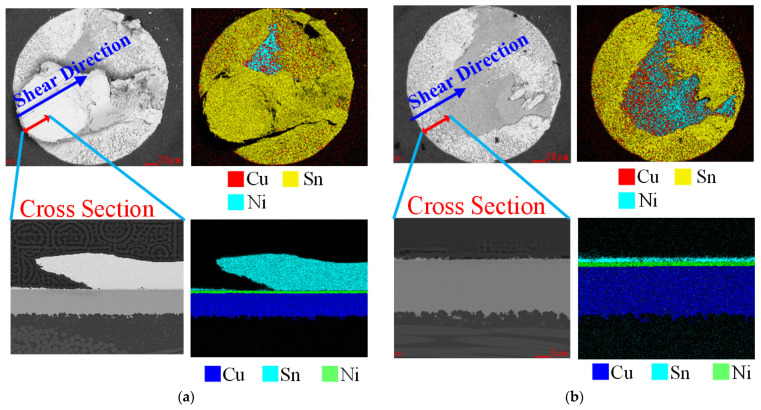
Surface, partial cross-section and chemical element distribution figures of two fracture modes: (**a**) solder bulk fracture; (**b**) solder/pad interface fracture.

By means of morphology contrast among all the ball fracture specimens, it was found that there are two types of fracture modes, namely, solder bulk fractures and solder/pad interface fractures. Bulk fracturing means that cracking occurs in the body of the solder ball, while interface fracturing implies relatively exhaustive ball stripping. Actually, these two kinds of ball fractures experience two different types crack initiating, namely, the expanding and final fracturing procedures, in ball shear tests. In the bulk fracture mode, the cracking begins in the body of the solder ball and expands along the shear direction inside the bulk material. After the shearing test, numerous apparent deep strip-type dimples emerge in the final fracture zone, which reflects the plastic fracture features. In the interface fracture mode, the cracking initiates just below the interface and expands along the surface, and the location of the final fracture zone shows great randomness. Of course, most of the final fracture zones are flat and smooth, with deep dimples rarely appearing in these zones. All these features imply the characteristics of brittle fracture [25,26]. The surface, partial cross section, and chemical element distribution figures of these two fracture modes are shown in Figure 7. As displayed s in the typical ball image in Figure 6, in the bulk facture mode fracturing occurs in the body of the ball and a block of Sn is left on the surface. Conversely, in the interface fracture mode the cracking location is situated at the ball/pad interface and the Sn ball is entirely cleared from the surface, which means that only area 1 and area 2 (see Figure 6) exist here.

**Figure 8:**
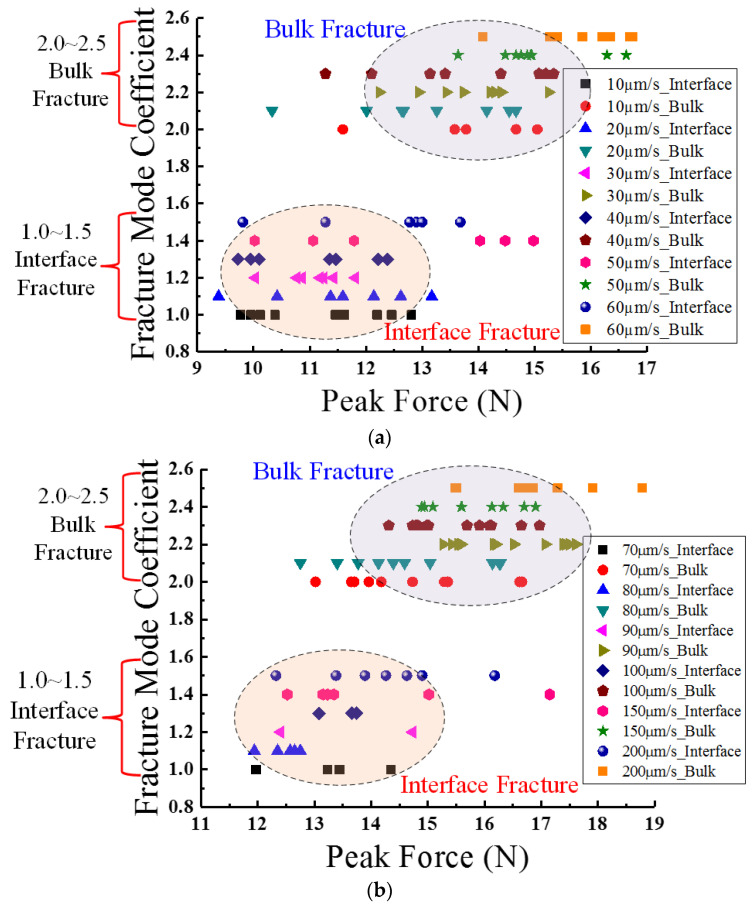
Statistical analysis results of peak force values according to the difference in fracture mode: (**a**) shear rates from 10 to 60 μm/s; (**b**) shear rates from 70 to 200 μm/s.

In addition, it was verified from the deformation feature analysis of the samples that the two typical fracture modes both appeared in each shearing speed test, and are an important factor that may determine the peak force level of different ball samples under the same shear rate. Figure 8 shows the statistical analysis results for all the peak force values according to the difference in fracture modes among ball samples. In these two figures the fracture mode coefficient is used to distinguish the two fracture modes; coefficient ranges from 1.0 to 1.5 indicate interface fracture, while ranges from 2.0 to 2.5 indicate bulk fracture. In each coefficient range, every 0.1 coefficient increment represents a shear speed variation.

As shown in Figure 8, the peak force values of bulk fracture balls are universally larger than those for the interface fracture balls under the same shear rate. The peak force focus region of bulk fractures is always located at the right side of the interface fracture focus region. In addition, the peak force distribution range in Figure 8a is 9 N to 17 N, while it is 11 N to 19 N in Figure 8b, verifying the previously-observed phenomenon that the mean peak force increases along with the increment of shear rate. Furthermore, it was discovered that the ratios of bulk fracture balls among all the samples increases with the shear rate as well, which indicates that interface fracture is more likely to happen in the relatively low-speed tests.

**Figure 9:**
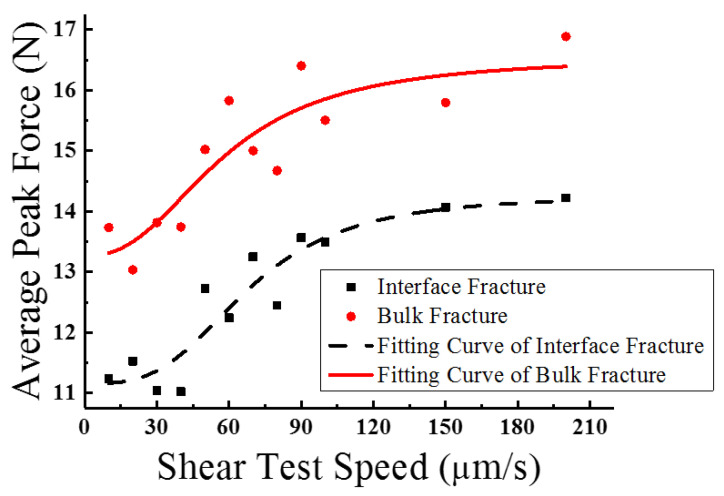
Average peak force distributions and fitting curves for the two fracture modes.

In order to analyze the diversity between the two fracture modes more clearly, the average peak force values of each speed and each mode were calculated and their respective variation trends were described, as shown in Figure 9.

Obviously, the peak force variation trends of the two fracture modes are extremely similar. Their approximation equations are

(2) $$F_{p\_I}=B_{1}+\frac{A_{1}-B_{1}}{1+{\left(\frac{v}{v_{1}}\right)}^{p_{1}}}$$

(3) $$F_{p\_B}=B_{2}+\frac{A_{2}-B_{2}}{1+{\left(\frac{v}{v_{2}}\right)}^{p_{2}}}$$

where $F_{p\_I}$ and $F_{p\_B}$ represent the average peak forces for interface and bulk fracture modes, v is the shear test speed, and $A_{1}$, $A_{2}$, $B_{1}$, $B_{2}$, $v_{1}$, $v_{2}$, $p_{1}$, and $p_{2}$ are the fitting factors; their typical values are shown in Table 1.

As exhibited in Figure 9, the two fitting curves both experience a gradual rise along with the increment of shear rates; it is obvious that the peak force curve of the bulk fracture mode is entirely above that of the interface fracture mode. The differences between them for the same test speeds are almost entirely within the range of 2 N to 2.5 N. As discussed above, the plastic deformation ability of solder materials is determined by the generating and moving capabilities of dislocations in the strain loading process. Distinctly, compared with solder bulk material, the interface section is a structure that is composed of large numbers of compounded material defects such as grain boundaries, intermetallic compounds, and dislocations. All these defects restrict the combining energies of the material interface and the connecting pads, leading to a higher plastic deformation ability in the interface section and a more easily-reached cracking property. Therefore, during a uniform shearing test it is much easier for fragmentation to occur at the material interface, and a relatively low shearing force is required to entirely strip the balls from the interface. In contrast, in bulk fractures the balls have greater adhesive power with the pads, and a higher combining energy must be overcome in order for fractures to occur. In this failure mechanism, it is believed that the bulk fracture balls enjoy higher reliability and stability under complex loading than those balls which are more likely to experience an interface fracture.

## 4. Conclusions

In order to investigate the mechanical properties and failure mechanisms of Sn96.5Ag3Cu0.5 solder structures, large numbers of low-speed ball shear tests based on a specific ball structure were conducted at continuous speeds from 10 μm/s to 200 μm/s. According to the experiments, it is obvious that all solders experience a full elasticity–plasticity deformation process. The deformation trends among solders in the elastic stage are consistent, while those in the plastic stage are extremely different. Based on the results of statistical analysis, the mechanical properties of solder balls are greatly related to shear speed. The mean peak force increases with the increment of speed, and the rise of the slope undergoes a great change from large to small. By utilizing SEM and ingredient analysis along with analysis of the cracking surface features, all the ball samples were classified into two types, namely, bulk fracture mode and interface fracture mode. It was proven that the bulk fracture balls have greater adhesive power with the connecting pads compared with the interface fracture ones. This fact may seriously affect the strain–deformation features and the reliability of solder balls. Thus, for accuracy considerations it is extremely important to distinguish between fracture modes and to utilize appropriate functions concerning a realistic application scenario.

## Figures and Tables

**Table 1:** Fitting factors for interface and bulk fracture modes.

Coefficient	$A_{1}$	$A_{2}$	$B_{1}$	$B_{2}$	$v_{1}$	$v_{2}$	$p_{1}$	$p_{2}$
**Typical Value**	11.16	13.27	14.27	16.55	67.93	57.97	3.21	2.42

## Data Availability

The data presented in this study are available in the supplementary material.

## Supplementary Materials

The following supporting information can be downloaded at: https://www.mdpi.com/article/10.3390/ma15072455/s1.
